# Human RECQ5 helicase promotes repair of DNA double-strand breaks by synthesis-dependent strand annealing

**DOI:** 10.1093/nar/gkt1263

**Published:** 2013-12-05

**Authors:** Shreya Paliwal, Radhakrishnan Kanagaraj, Andreas Sturzenegger, Kamila Burdova, Pavel Janscak

**Affiliations:** ^1^Institute of Molecular Cancer Research, University of Zurich, CH-8057 Zurich, Switzerland and ^2^Institute of Molecular Genetics, Academy of Sciences of the Czech Republic, 14300 Prague, Czech Republic

## Abstract

Most mitotic homologous recombination (HR) events proceed via a synthesis-dependent strand annealing mechanism to avoid crossing over, which may give rise to chromosomal rearrangements and loss of heterozygosity. The molecular mechanisms controlling HR sub-pathway choice are poorly understood. Here, we show that human RECQ5, a DNA helicase that can disrupt RAD51 nucleoprotein filaments, promotes formation of non-crossover products during DNA double-strand break-induced HR and counteracts the inhibitory effect of RAD51 on RAD52-mediated DNA annealing *in vitro* and *in vivo*. Moreover, we demonstrate that RECQ5 deficiency is associated with an increased occupancy of RAD51 at a double-strand break site, and it also causes an elevation of sister chromatid exchanges on inactivation of the Holliday junction dissolution pathway or on induction of a high load of DNA damage in the cell. Collectively, our findings suggest that RECQ5 acts during the post-synaptic phase of synthesis-dependent strand annealing to prevent formation of aberrant RAD51 filaments on the extended invading strand, thus limiting its channeling into potentially hazardous crossover pathway of HR.

## INTRODUCTION

DNA double-strand break (DSB) is the most dangerous type of DNA damage because its inaccurate repair can lead to chromosomal rearrangements, a hallmark of tumorigenesis and tumor progression. In eukaryotic cells, two mechanistically distinct pathways are known to efficiently repair DNA DSBs: non-homologous end joining and homologous recombination (HR). HR is mainly restricted to S phase, peaking in mid-S, and requires an intact homologous sequence to be used as a repair template (1–3). It is initiated by nuclease-mediated resection of the DNA ends to generate 3′-single-stranded (ss) DNA tails that are coated by the ssDNA-binding protein RPA (4). In the next step, the RAD51 recombinase replaces RPA on these ssDNA tails with the help of mediators such as BRCA2 to form a nucleoprotein filament that catalyzes the invasion of the donor chromatid, giving rise to a three-stranded structure called the displacement (D)-loop (1). After DNA synthesis primed by the invading strand, repair can proceed *via* two main sub-pathways referred to as the canonical DSB repair (DSBR) and synthesis-dependent strand annealing (SDSA) (1,2). In DSBR pathway, the second DNA end is captured by the D-loop to form an intermediate with two Holliday junctions, referred to as double Holliday junction (dHJ). This joint DNA molecule can be either resolved by specialized endonucleases into crossover (CO) or non-crossover (NCO) products or dissolved by the BLM-TOPOIIIα-RMI1/2 (BTR) complex, which gives rise exclusively to NCO products (5–7). In the SDSA pathway, the extended D-loop is disrupted by a DNA helicase, and the newly synthesized DNA is annealed to the ssDNA tail of the other part of the broken chromosome, which is followed by gap-filling DNA synthesis and ligation. As a result, SDSA yields exclusively NCO products (8).

The HR sub-pathways are under strict regulation to select the most appropriate outcome in a given state of the cell (2,9). Although formation of COs is favored during meiosis to ensure genetic diversity and accurate chromosome segregation, it is suppressed in mitotic cells to prevent loss of heterozygosity and chromosomal translocations (10,11). Recent studies in yeast and mammalian cells suggest that HJ resolvases are active only during mitosis, biasing the outcome of recombination toward NCO products while also ensuring the elimination of any persistent joint DNA molecules (11). Most NCOs arising during HR-mediated DSBR are produced by SDSA rather than by the canonical DSBR pathway (12). Moreover, the resolution of HJs is highly constrained to generate CO products (12). Thus, it appears that the SDSA mechanism is preferred over DSBR in mitotic cells.

In budding yeast, the Mph1 DNA helicase suppresses COs by acting in a pathway distinct from dHJ dissolution (13). Mph1 influences outcome rather than the efficiency of recombinational repair events, suggesting that it acts by shunting a DNA repair intermediate into the SDSA pathway (13). In support of this notion, biochemical evidence indicates that Mph1 is capable of disrupting Rad51-made D-loops (13). Another suppressor of COs in yeast proposed to act via promotion of SDSA is Srs2, an UvrD-type DNA helicase that has the capacity to displace Rad51 from ssDNA (14,15). The mechanism of CO suppression by Srs2 appears to differ from that of Mph1. Cells lacking Srs2 display a failure to complete ectopic gene conversion with NCO outcome, which reduces the overall repair efficiency, and therefore increases the proportion of CO products among completed recombination events (14). Although Srs2 can unwind DNA duplexes covered by Rad51, it fails to unwind Rad51-made D-loops (13,16). Instead, the anti-recombinase activity of Srs2 *in vivo* is dependent on its ability to bind RAD51, suggesting that Srs2 might promote SDSA by regulating Rad51 filament stability (17).

The closest sequence homolog of Srs2 in mammals and other vertebrates is FBH1, which is also found in fission yeast but not in budding yeast. Several lines of *in vivo* evidence suggest that this UvrD-type helicase regulates HR at the stage of RAD51 filament assembly, but its role in SDSA is yet to be assessed (18). Another potential ortholog of Srs2 in mammals is RECQ5, which belongs to RecQ family of DNA helicases (19). Biochemical studies have shown that RECQ5 binds directly to RAD51 and possesses the ability to disrupt the ATP-bound form of RAD51-ssDNA filament in a manner dependent on its ssDNA-translocase activity and interaction with RAD51 (20,21). In accordance with this finding, phenotypic analysis of chicken and mouse knockout cells have revealed that RECQ5 regulates HR to suppress the formation of COs (20,22,23). Moreover, a recent study using chicken DT40 cells has demonstrated that RECQ5 suppresses COs in a manner dependent on its interaction with RAD51 (24). Here we provide several lines of evidence suggesting that RECQ5 promotes SDSA by disrupting aberrant RAD51-ssDNA filaments formed during the post-synaptic stage of HR.

## MATERIALS AND METHODS

### Antibodies and siRNAs

All antibodies and siRNAs used in this study are described in Supplementary Materials and Methods.

### HR and SSA reporter assays

Maintenance of reporter cell lines (HEK293/DR-GFP, U2OS/DR-GFP, HEK293/SA-GFP or U2OS/SA-GFP), culture conditions and FACS analysis were done as described previously (25,26). Cells were seeded in a poly-lysine-coated 6-well plate at a density of 0.6 × 10^6^ cells per well and transfected 24 h later with appropriate siRNA (40 nM). After 24 h, 0.2 × 10^6^ cells for each siRNA tested were seeded in a 12-well plate, and a day later transfected with 0.6 μg of the I-SceI-expressing plasmid pCBASce (27) or empty vector (pcDNA3.1) using JETprime (Polyplus) for HEK293 cells or Lipofectamine 2000 (Invitrogen) for U2OS cells according to the manufacturer’s instructions. After 6 h, cells were again transfected with appropriate siRNA (15 nM), and 48–72 h later, GFP-positive cells were quantified by flow cytometry on a Cyan ADP (Dako) using Summit software (Beckman Coulter). For overexpression of RECQ5 in U2OS/SA-GFP cells, a pcDNA3.1/HisC-based vector expressing the wild-type or mutant forms of RECQ5 (0.6 μg) was co-transfected with pCBASce (0.6 μg) using Lipofectamine 2000 (21). Cells were subjected to FACS analysis 72 h after transfection.

### Strand-annealing assays

Proteins used for strand-annealing assays were purified as described in Supplementary Materials and Methods. All reactions were carried out at 30°C in buffer R [20 mM Tris-acetate (pH 7.9), 50 mM potassium acetate, 10 mM magnesium acetate and 1 mM DTT] supplemented with ATP-regenerating system (10 U/ml creatine phosphokinase and 12 mM phosphocreatine). The 5 nM 5′-^32^P-labeled f9 oligonucleotide (59-mer) was pre-incubated for 3 min with or without 200 nM RAD51^K133R^ in a final volume of 20 μl (reaction A). In a separate tube, 5 nM f7 oligonucleotide (30-mer), complementary to 5′ end of f9, was mixed with 120 nM RAD52 and 40 nM RPA in a final volume of 20 μl (reaction B). Where required, the latter reaction mixture also contained 5 nM 59-bp DNA duplex prepared by annealing of unlabeled f9 and f9-C oligonucleotides. The reactions A and B were mixed together, and where required RECQ5, RECQ5^K58R^, RECQ5^Δ652^^−674^, WRN or FBH1 were added to a final concentration of either 80 or 40 nM. In all, 5 μl aliquots were removed at the indicated time points and mixed with 2.5 µl of stop solution [125 nM f9 (unlabeled), 33% (v/v) glycerol, 1% (w/v) SDS, 0.15 M EDTA, 0.5 mg/ml proteinase K and 0.1% (w/v) Bromophenol Blue] followed by a 5 min incubation at 30°C. Samples were subjected to electrophoresis in a 10% non-denaturing PAGE run at 100 V for 2 h in 1× TBE buffer. Radiolabeled DNA species were visualized by phosphorimaging and quantified using ImageQuant TL software.

### Chromatin immunoprecipitation assay

Chromatin immunoprecipitation (ChIP) experiments were done with the ChIP-IT® Express kit (Active Motif) as described previously (28). Briefly, U2OS/DR-GFP cells were seeded in a 10-cm plate and transfections of siRNA and I-SceI plasmid were performed as described for DR-GFP reporter assays. Two days after I-SceI plasmid transfection, cells were cross-linked with formaldehyde [1% (v/v)] at room temperature for 10 min, followed by addition of glycine (125 mM) to quench the cross-linking reaction. Chromatin fragments used in immunoprecipitation reactions were prepared by shearing of cross-linked chromatin using a bioruptor sonication device (Diagenode). One tenth of the sonicated chromatin was stored at −70°C to be used as an input sample. For each ChIP reaction, sonicated chromatin (8 µg) was immunoprecipitated overnight at 4°C with either anti-RAD51 antibody or control IgG (4 µg each) and protein G-coated magnetic beads (Active Motif). After washing, immunocomplexes were eluted from the beads and de-cross-linked according to the manufacturer’s instructions (Active Motif). ChIPed and input samples were purified with the QIAquick PCR Purification Kit (Qiagen), and DNA was eluted with 50 µl of water. At least two independent experiments were performed for each ChIP reaction. In each case, eluted DNA sample (2–3 µl) was subjected to quantitative real-time PCR (qPCR) analysis in hexaplicate on a Roche LightCycler 480 Real-time PCR system with the use of Roche LightCycler 480 DNA SYBR Green I master. Data were analyzed using the Pfaffl’s method (29). Fold enrichment of RAD51 binding on each target region was calculated as a ratio of the amount of DNA estimated for the RAD51-specific antibody versus the amount of DNA estimated for the control IgG. Primers used in ChIP-qPCR assays are described in Supplementary Materials and Methods.

### Sister chromatid exchange assay

Sister chromatid exchange (SCE) assay was done as described previously (30). Details are provided in Supplementary Materials and Methods.

## RESULTS

### RECQ5 promotes formation of non-crossover products during DSB-induced HR in human cells

**Figure 1.**
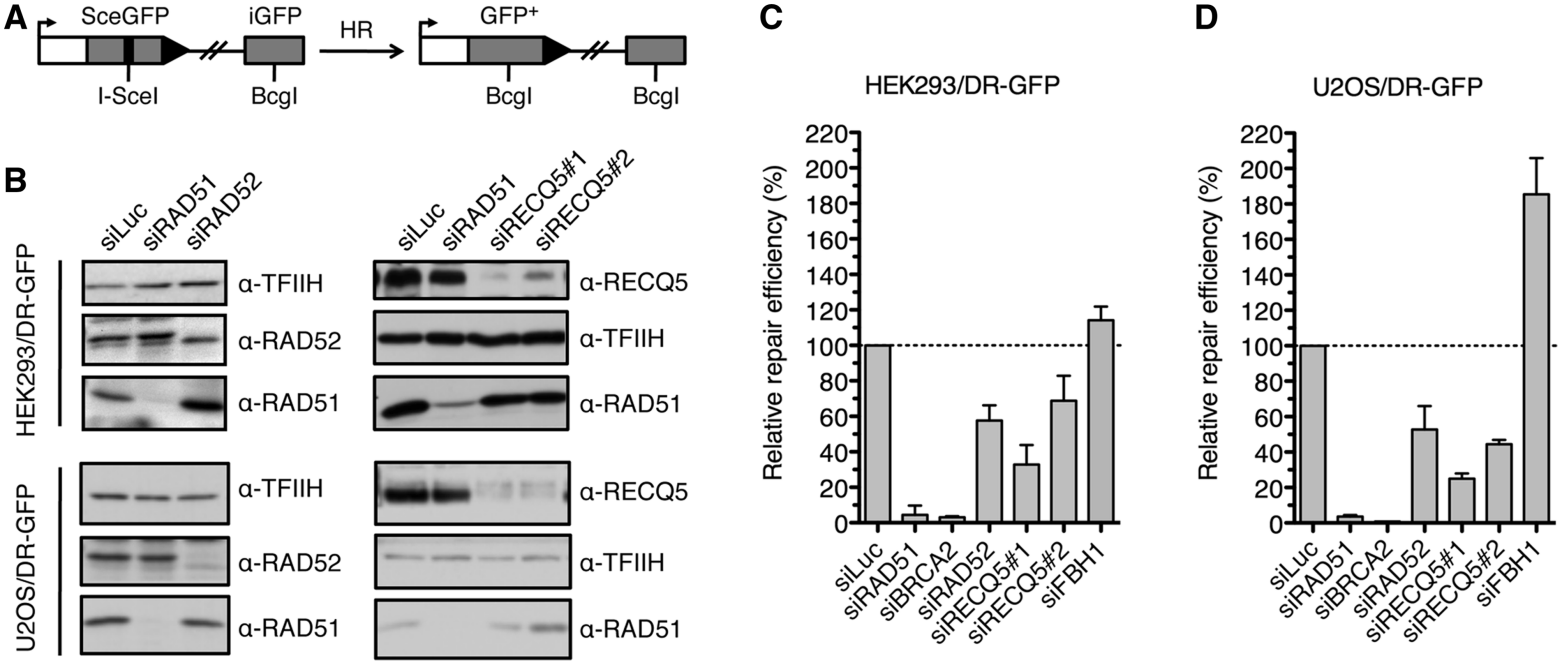
RECQ5 promotes homologous recombination with non-crossover outcome. (**A**) Scheme of the DR-GFP reporter cassette. The site-specific DSB in the reporter cassette is generated by I-SceI endonuclease. Only NCO events give rise to a functional GFP allele. (**B**) Western blot analysis of extracts from HEK293/DR-GFP and U2OS/DR-GFP cells transfected with indicated siRNAs. Blots were probed with indicated antibodies. (**C**) Efficiency of HR-mediated repair of I-SceI-induced DSB in HEK293/DR-GFP cells treated with indicated siRNAs. Cells were transfected with appropriate siRNA (40 nM) two days before transfection of I-SceI-expressing plasmid. Percentage of GFP-positive cells was measured by flow cytometry 2 days after DSB induction and taken as a measure of DSBR efficiency. Values plotted represent relative repair efficiency calculated as a percentage of repair efficiency measured in cells transfected with control siRNA (siLuc; 100%). All data points represent an average of at least three replicates with error bars indicating standard deviation. (**D**) Efficiency of HR-mediated repair of I-SceI-induced DSB in U2OS/DR-GFP cells treated with indicated siRNAs as compared with cells transfected with control siRNA. Experiments were performed as in (C) except that the flow cytometry analysis was performed 3 days after I-SceI transfection. HR, homologous recombination; DSB, double-strand break; NCO, non-crossover; and GFP, green fluorescent protein.

To gain deeper insight into the molecular mechanism of SDSA in mammalian cells, we investigated the role of two potential human orthologs of Srs2, namely, FBH1 and RECQ5, in the formation of NCO products during repair of endonuclease-induced DSBs. To selectively detect NCO events, we used the established reporter cell lines HEK293/DR-GFP and U2OS/DR-GFP (25,26,31). The DR-GFP reporter consists of a direct repeat of two mutated GFP alleles: a full length GFP interrupted by a recognition site for the I-SceI endonuclease and an internal GFP fragment that serves as a donor for HR-mediated repair of a DSB created by I-SceI in the proximal GFP allele (Figure 1A). HR-mediated repair of this DSB *via* an NCO event gives rise to a functional GFP allele, whereas repair by crossing over yields a C-terminally truncated GFP allele that does not encode for a fluorescent protein. Thus, quantification of GFP-positive cells by flow cytometry provides a measure of NCO repair efficiency. Proteins of interest were depleted from the DR-GFP reporter cell line by transfection of small interfering RNAs (siRNAs) (Figure 1B and Supplementary Figure S1). Cells were subsequently transfected with an I-SceI expression vector to induce a DSB in the reporter cassette, and the percentage of GFP positive cells arising upon DSBR was measured 2–3 days after plasmid transfection. As expected, formation of a functional GFP allele was impaired in cells depleted for the RAD51 recombinase or its loader BRCA2 (Figure 1C and D). Repair efficiency also significantly decreased on depletion of RAD52 that mediates DNA strand annealing in a reaction stimulated by RPA and is required for SDSA in budding yeast (Figure 1C) (32,33). Interestingly, depletion of BLM, the key component of the BTR complex, led to a significant increase in repair efficiency relative to control, indicating that dHJ dissolution has no role in formation of NCO repair products in the DR-GFP system (Supplementary Figure S2). This is consistent with the proposal that limited regions of homology, as is the case during ectopic recombination, decrease the possibility of forming a dHJ structure due to impediment of strand exchange by DNA end resection beyond the homologous region (34). This would imply that SDSA accounts for the majority of NCO repair events induced by I-SceI in cells harboring the DR-GFP cassette. BLM helicase probably disrupts joint DNA molecules formed during the repair process.

As for the putative Srs2 orthologs tested, only depletion of RECQ5 led to a marked reduction of repair efficiency both in HEK293 and U2OS cells, without any significant changes in cell cycle distribution (Figure 1C and D and Supplementary Figure S3). RECQ5 depletion also dramatically reduced the elevated NCO repair efficiency in HEK293/DR-GFP cells lacking BLM (Supplementary Figure S2). On the contrary, depletion of FBH1 led to a significant elevation of repair efficiency particularly in U2OS cells (Figure 1C and D). Thus, our results suggest that RECQ5 might promote DNA DSBR by the SDSA pathway of HR, whereas FBH1 might act as an SDSA suppressor.

### RECQ5 counteracts the inhibitory effect of RAD51 on DSBR by SSA in human cells

**Figure 2.**
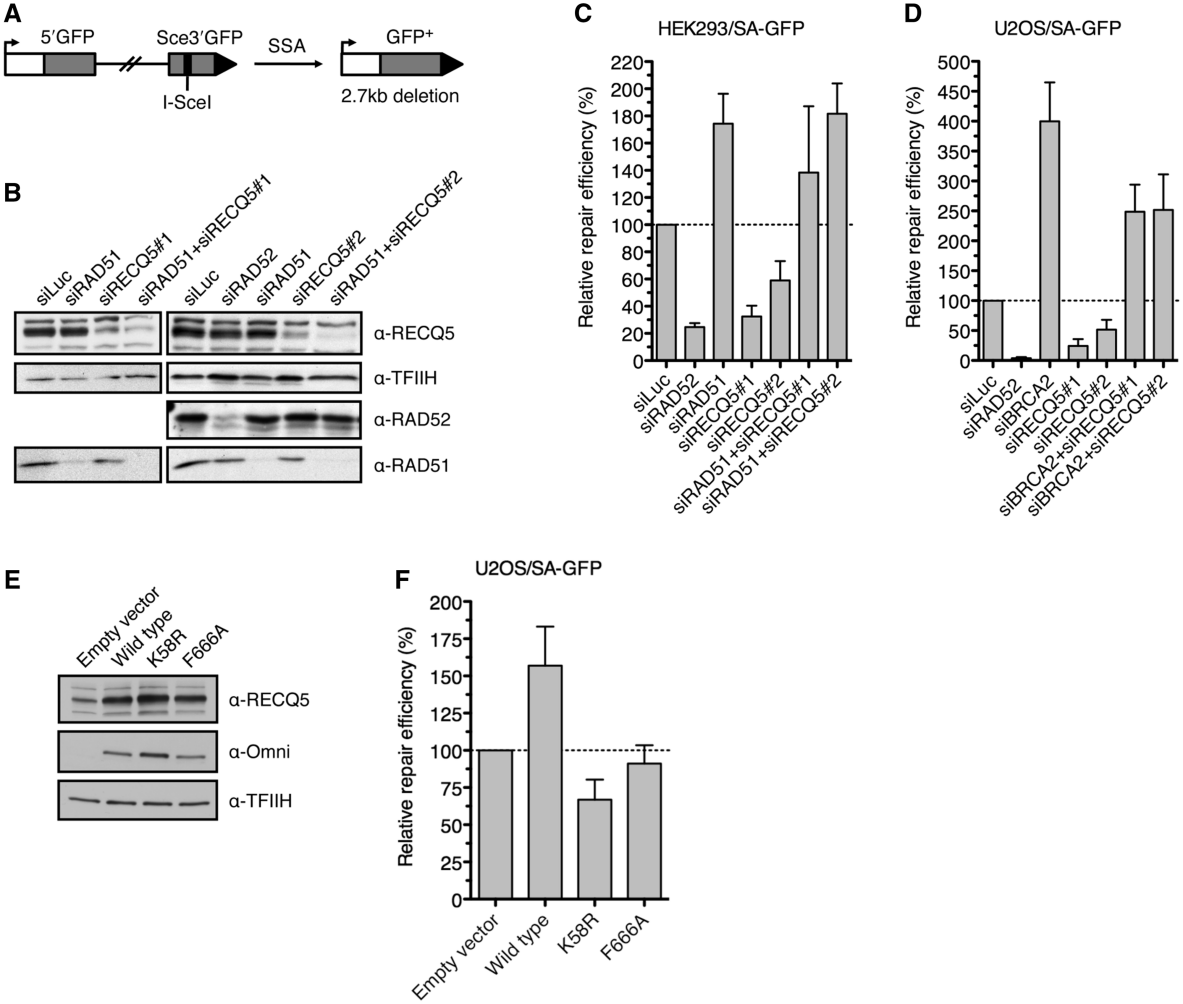
RECQ5 suppresses inhibitory effect of RAD51 on DNA DSBR by SSA. (**A**) Scheme of the SA-GFP reporter cassette. SSA-mediated repair of I-SceI-generated DSB results in the formation of a functional GFP allele. (**B**) Western blot analysis of extracts from HEK293/SA-GFP cells transfected with indicated siRNAs. The blots were probed with indicated antibodies. (**C**) Efficiency of SSA-mediated repair of I-SceI-induced DSB in HEK293/SA-GFP cells transfected with indicated siRNAs. Cells were transfected with appropriate siRNA (40 nM) 2 days before transfection of I-SceI-expressing plasmid. Percentage of GFP-positive cells was determined by flow cytometry 2 days after DSB induction and taken as a measure of repair efficiency. Values plotted represent relative repair efficiency calculated as a percentage of repair efficiency measured in cells transfected with control siRNA (siLuc; 100%). All data points represent an average of at least three replicates with error bars indicating standard deviation. (**D**) Efficiency of SSA-mediated repair of I-SceI-induced DSB in U2OS/SA-GFP cells transfected with indicated siRNAs. Experiments were performed as in (C) except that the percentage of GFP-positive cells was determined 3 days after DSB induction. (**E**) Western blot analysis of extracts from U2OS/SA-GFP cells transfected with pcDNA3.1/HisC-based vectors expressing wild-type RECQ5 or its mutants, K58R and F666A, respectively, as fusions with an Omni-tag. The blots were probed with indicated antibodies. (**F**) Effect of overexpression of the wild-type and mutant forms of RECQ5 on the efficiency of SSA-mediated repair of I-SceI-induced DSB in U2OS/SA-GFP**.** Cells were transfected with appropriate RECQ5 expression vector in combination with the plasmid expressing I-SceI. Percentage of GFP-positive cells was determined 3 days after plasmid transfection. Values plotted represent relative repair efficiency calculated as a percentage of repair efficiency measured in cells transfected with empty vector. All data points represent an average of at least three replicates with error bars indicating standard deviation. SSA, single-strand annealing; DSB, double-strand break; and GFP, green fluorescent protein.

It is possible that RECQ5 promotes SDSA by catalyzing disruption of an aberrant RAD51 filament that might form on the newly synthesized DNA strand after unwinding of the extended D-loop. This filament would inhibit the ssDNA-annealing step of SDSA and could promote reformation of the D-loop, shifting the balance between the HR sub-pathways in favor of DSBR. To explore this possibility, we used a GFP-based reporter for DSBR by single-strand annealing (SSA), which mechanistically resembles the post-synaptic phase of SDSA (25,31). The reporter cassette, termed SA-GFP, contains two directly oriented GFP gene fragments, 5′GFP and Sce3′GFP, forming a 280-bp repeat (Figure 2A). SSA-mediated recombination between the repeated sequences triggered by an I-SceI-generated DSB in the distal GFP fragment results in the formation of a functional GFP gene. Using HEK293-based SA-GFP reporter system (HEK293/SA-GFP), we found that depletion of RAD51 or its loader BRCA2 resulted in a marked increase (2- to 4- fold) in SSA repair efficiency compared with control, providing evidence that formation of RAD51 filaments on resected DNA ends inhibits DSBR by SSA in human cells (Figure 2B and C and Supplementary Figure S4). On the contrary, depletion of RAD52 protein resulted in a dramatic decrease in the frequency of SSA repair events, which is consistent with the proposed role for RAD52 in promoting DNA annealing during SSA (Figure 2B and C) (31). We repeated our experiments with U2OS/SA-GFP cells, and we found that knockdown of RAD51 resulted in lethality. However, cells depleted for BRCA2 were viable and showed a dramatic increase in SSA repair efficiency compared with control cells, further supporting the aforementioned proposal (Figure 2D). As shown with the HEK293/SA-GFP cells, knockdown of RAD52 in U2OS/SA-GFP cells dramatically impaired SSA repair efficiency (Figure 2D).

Depletion of RECQ5 caused a significant reduction in SSA repair efficiency, but only if the cells contained RAD51 (HEK293/SA-GFP cells) and BRCA2 (HEK293/SA-GFP and U2OS/SA-GFP cells) (Figure 2 and Supplementary Figure S4). HEK293/SA-GFP cells depleted for both RECQ5 and RAD51 displayed a SSA repair capacity that was similar to that of cells depleted for RAD51 alone (Figure 2B and C). Similarly, the SSA defect caused by lack of RECQ5 was rescued by co-depletion of BRCA2 (Figure 2D and Supplementary Figure S4). Thus, these data suggest that RECQ5 counteracts the inhibitory effect of RAD51 on DSBR by SSA, most likely by catalyzing disruption of RAD51 filaments formed on ssDNA generated by DNA-end resection.

To verify this hypothesis, we compared the effects of overexpression of wild-type and mutant forms of RECQ5 on the efficiency of SSA-mediated repair of I-SceI-induced DSBs in U2OS/SA-GFP cells. The following RECQ5 mutants were tested: (i) mutant containing a K58R substitution in the ATP-binding site of RECQ5, which abolishes the ATPase and helicase activities of the enzyme; and (ii) mutant containing an F666A substitution in the RAD51-binding domain of RECQ5, which impairs RECQ5-RAD51 complex formation (21,35). Both mutants are defective in catalyzing RAD51 filament disruption (20,21). In line with the proposed model, we found that overexpression of wild-type RECQ5 in U2OS/SA-GFP cells resulted in a marked increase in SSA repair efficiency as compared with cells harboring control vector (Figure 2E and F). In contrast, the K58R mutant of RECQ5, which is proficient in binding to RAD51, exhibited a significant trans-dominant negative effect on the repair efficiency, possibly by preventing the access of the endogenous RECQ5 protein to RAD51 filaments formed on resected DNA ends (Figure 2E and F). For the F666A mutant of RECQ5, we found that its overexpression had no effect on SSA-mediated DSBR in U2OS/SA-GFP cells, which is consistent with the inability of this mutant to interact with RAD51 filaments (Figure 2E and F). Thus, our data strongly suggest that RECQ5 enhances the efficiency of SSA-mediated DSBR by disrupting RAD51-ssDNA filaments formed at resected DNA ends.

### RECQ5 counteracts the inhibitory effect of RAD51 on RAD52-mediated DNA annealing *in vitro*

**Figure 3.**
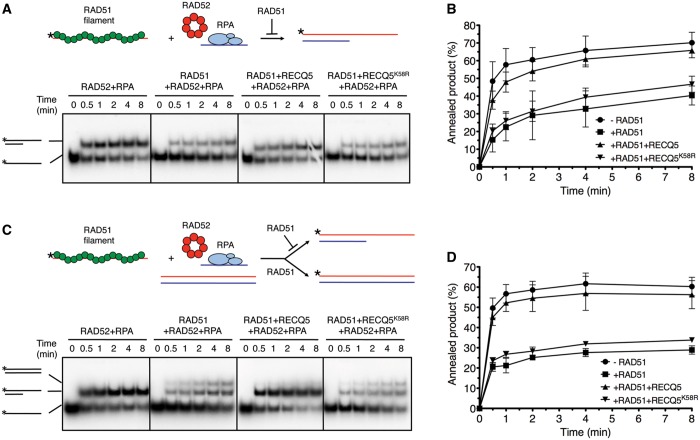
RECQ5 helicase counteracts the inhibitory effect of RAD51 on RAD52-mediated ssDNA annealing *in vitro*. (**A**) Upper panel: reaction scheme depicting the effect of RAD51 (green circles) on annealing of two complementary oligonucleotides (59-mer and 30-mer represented by red and blue lines, respectively) in presence of RAD52 and RPA. RAD52 is depicted as a heptameric ring structure (red circles). The 30-mer oligonucleotide can accommodate binding of one RPA heterotrimer (light blue ovals). Lower panel: all reactions were carried out at 30°C in buffer R supplemented with ATP-regenerating system. Reactions contained 5′ end radiolabeled 59-mer oligonucleotide (2.5 nM), either free or pre-coated with RAD51^K133R^ (100 nM), a 30-mer oligonucleotide (2.5 nM) complementary to the 5′-half of the 59-mer, RAD52 (60 nM) and RPA (30 nM). Where indicated, RECQ5 or RECQ5^K58R^ were present at a concentration of 80 nM. Reaction aliquots at indicated time points were subjected to PAGE followed by phosphorimaging as described in ‘Materials and Methods’. (**B**) Quantification of data shown in (A). Each data point represents the mean of three independent experiments. Error bars represent standard deviation. (**C**) Upper panel: reaction scheme depicting the effect of RAD51 on annealing of two complementary oligonucleotides in presence of a homologous duplex, RAD52 and RPA. RAD51 filament formed on the radiolabeled oligonucleotide (red line with asterisk) inhibits RAD52/RPA-mediated annealing and promotes strand exchange with the homologous duplex. Lower panel: reactions were carried out and analyzed as in (B). Homologous 59-mer duplex was present at a concentration of 2.5 nM. Schemes of radiolabeled DNA species are shown on left. Radioactive label at the 5′ end is depicted by asterisks. (**D**) Quantification of data shown in (C). Each data point represents the mean of three independent experiments. Error bars represent standard deviation.

Next, we performed biochemical experiments addressing the effect of RAD51 on RAD52-mediated annealing of two complementary oligonucleotides either in the absence or in the presence of RECQ5. In these assays, we used an ATP hydrolysis-deficient mutant of RAD51, RAD51^K133R^, which can form a stable nucleoprotein filament in the presence of ATP, mimicking the *in vivo* ATP-bound form of the filament that is capable of catalyzing DNA strand exchange (20). Before annealing reactions, a 30-mer oligonucleotide was pre-incubated with RPA to form an ssDNA-RPA complex, whereas the other oligonucleotide (59-mer, radioactively labeled at its 5′ end) was pre-incubated either with RAD51^K133R^ to form a nucleoprotein filament or with the reaction buffer alone. We found that addition of RPA-coated 30-mer oligonucleotide to free 59-mer oligonucleotide in presence of RAD52 resulted in rapid formation of partial DNA duplex structure with a 3′-tail (Figure 3A, *first panel from the left*, and Supplementary Figure S5). However, this RAD52-mediated ssDNA annealing was impaired if the 59-mer oligonucleotide was pre-coated with RAD51^K133R^ before its addition to the annealing reaction, demonstrating that formation of RAD51-ssDNA filaments inhibits ssDNA annealing by RAD52 (Figure 3A and B). Similar results were obtained with wild-type RAD51, although the observed inhibitory effect was less pronounced than that of RAD51^K133R^ (Supplementary Figure S6). Remarkably, the inhibitory effect of RAD51^K133R^ on RAD52-mediated ssDNA annealing was almost completely lost on addition of RECQ5 to the reaction (Figure 3A and B). On the contrary, the helicase-deficient mutant of RECQ5 (RECQ5^K58R^) or a RECQ5 mutant lacking the RAD51-interacting domain (RECQ5^Δ652^^−674^) did not alleviate this inhibitory effect, suggesting that RECQ5 stimulated the ssDNA-annealing reaction by disrupting RAD51^K133R^-ssDNA filaments (Figure 3A and B and Supplementary Figure S7). In addition, we found that the inhibition of RAD52-mediated ssDNA annealing by RAD51^K133R^ was not relieved if RECQ5 was substituted by WRN RecQ helicase or by FBH1, indicating that this reaction is specific for RECQ5 (Supplementary Figure S7).

To substantiate the aforementioned findings, ssDNA annealing reactions were also carried out in presence of a homologous DNA duplex (59-mer), conditions resembling the post-synaptic phase of SDSA. We found that this DNA duplex had no effect on RAD52-mediated annealing of the two complementary oligonucleotides (Figure 3C and D). However, if the 59-mer oligonucleotide was pre-coated with RAD51^K133R^, we again observed a strong inhibition of RAD52-mediated ssDNA annealing with concomitant appearance of radioactively labeled 59-mer oligoduplex, an indicative of strand exchange reaction (Figure 3C and D). On addition of RECQ5, RAD51^K133R^-catalyzed strand exchange was inhibited, and RAD52-mediated ssDNA annealing was restored to a level detected in absence of RAD51^K133R^ (Figure 3C and D). Again this effect was not seen with the K58R mutant of RECQ5 (Figure 3C and D). These data suggest that RAD51 can promote reformation of D-loop during the post-synaptic phase of SDSA and that RECQ5 can counteract this reverse reaction by removing RAD51 from the invading strand.

### RECQ5 deficiency is associated with increased occupancy of RAD51 on DNA sequences flanking a DSB

**Figure 4.**
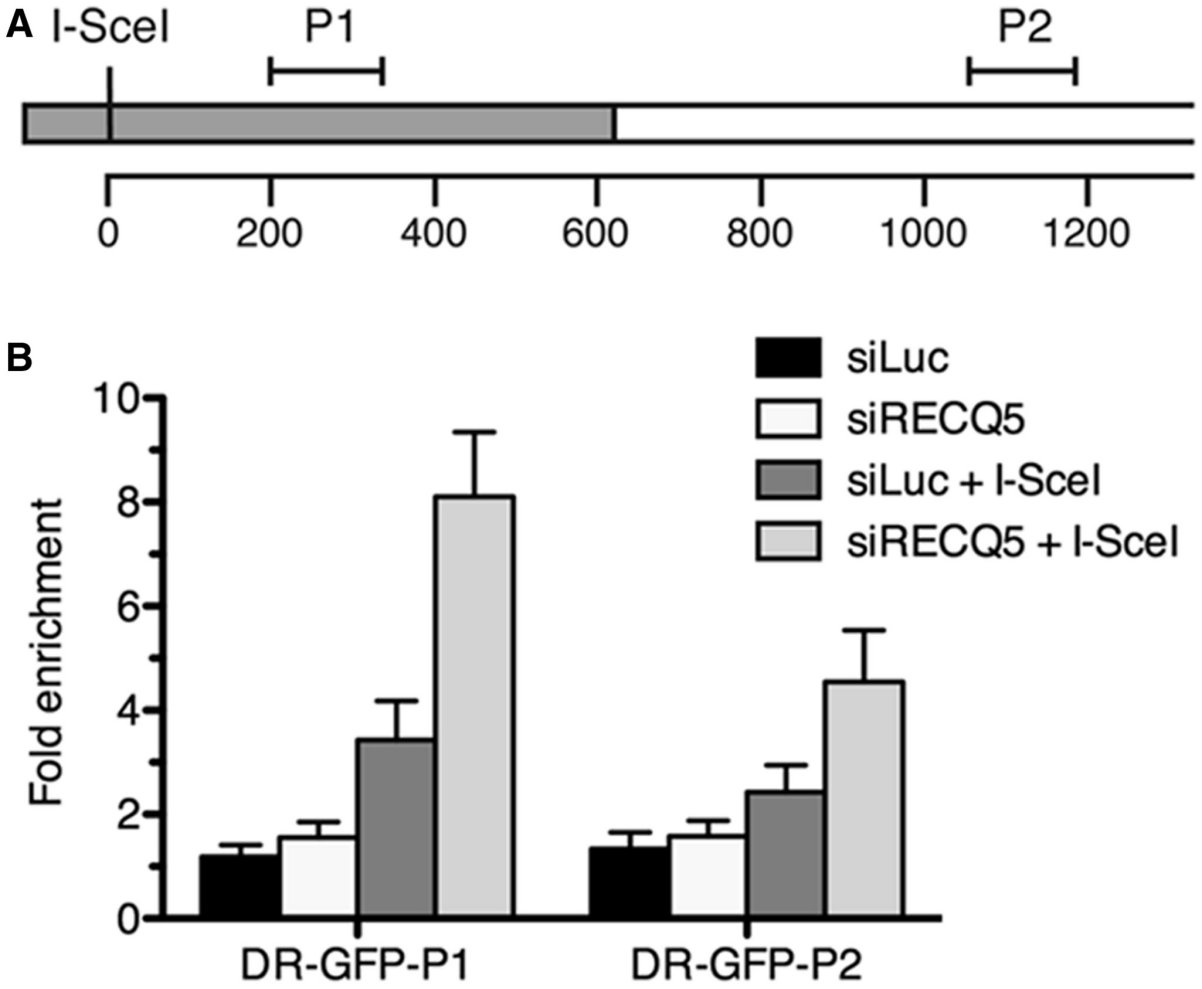
Effect of RECQ5 deficiency on RAD51 occupancy at chromatin flanking the I-SceI-induced DSB in U2OS/DR-GFP cells. (**A**) A schematic diagram of a part of the DR-GFP reporter cassette showing the locations of the regions amplified in ChIP-qPCR assays (P1 and P2). The GFP open reading frame with a single I-SceI recognition site is shown as gray box. The numbers correspond to base pairs. (**B**) Plot of ChIP-qPCR data. Mock-depleted (siLuc) and RECQ5-depleted (siRECQ5#1) cells were transfected with either I-SceI expression vector (+I-SceI) or empty vector, respectively. Chromatin for ChIP analysis was prepared 2 days after I-SceI transfection. Fold enrichment was calculated as a ratio of RAD51 antibody signal versus control IgG. ChIP, chromatin immunoprecipitation; and qPCR, quantitative real-time PCR.

To prove that the lack of RECQ5 is associated with persistence of RAD51 filaments on resected DNA ends during HR, we used ChIP to evaluate the effect of RECQ5 knockdown on RAD51 occupancy at chromatin flanking the I-SceI site in U2OS/DR-GFP cells two days after I-SceI plasmid transfection. Immunoprecipitated DNA fragments were subjected to quantitative real-time PCR analysis using primers amplifying the regions located downstream of I-SceI recognition sequence: P1, +181 to +325 and P2, +1037 to +1172 (Figure 4A). We found that RECQ5 knockdown in U2OS/DR-GFP cells was accompanied by a marked increase in the abundance of RAD51 localized near the I-SceI cutting site as compared with mock-depleted cells (Figure 4B). No significant binding of RAD51 to chromatin flanking the I-SceI site was detected in absence of I-SceI expression with both RECQ5-proficient and RECQ5-deficient cells. Thus, these data provide direct evidence that RECQ5 regulates RAD51 filament formation at DNA DSB sites.

### RECQ5 acts as CO suppressor in human cells

**Figure 5.**
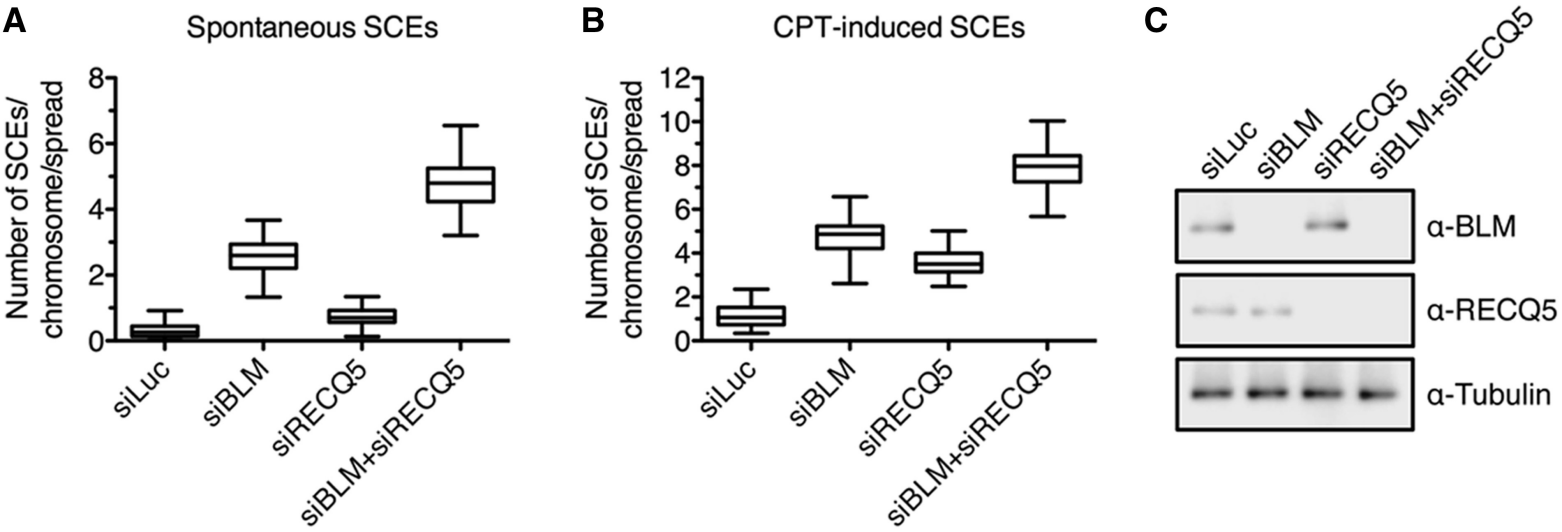
RECQ5 and BLM act in different pathways to suppress crossovers in human cells. (**A**) Frequency of spontaneous SCEs in U2OS cells transfected with indicated siRNAs. (**B**) Frequency of CPT-induced SCEs in U2OS cells transfected with indicated siRNA. Cells were treated with 40-nM CPT for 20 h where indicated. SCE assay and analysis was conducted as described in ‘Materials and Methods’. Each data point represents number of SCEs per chromosome in a single metaphase spread. The 50 metaphase spreads were analyzed from each condition. (**C**) Western blot analysis of extracts from U2OS cells transfected with indicated siRNAs. Blots were probed with indicated antibodies. SCE, sister chromatid exchange; and CPT, camptothecin.

To assess the role of RECQ5 in suppression of mitotic COs, we investigated the effect of siRNA-mediated depletion of RECQ5 and BLM on the frequency of SCEs in U2OS cells before and after induction of DNA DSBs by camptothecin (CPT). We observed that RECQ5 depletion in untreated cells had no significant effect on the SCE frequency, whereas depletion of BLM increased the SCE frequency by almost 3-fold compared with control cells (Figure 5A and C). Cells depleted for RECQ5 and BLM showed a much higher SCE frequency than cells depleted for BLM alone (Figure 5A and C), which is consistent with the studies in chicken and mouse cells (22,23). Importantly, on CPT treatment, a marked elevation of SCE frequency was observed not only in BLM-deficient cells but also in RECQ5-deficient cells, suggesting that RECQ5 has a role in CO suppression even in the presence of BLM if the load of DNA damage exceeds a certain threshold (Figure 5B). Again cells depleted for both RECQ5 and BLM exhibited a much higher frequency of CPT-induced SCEs than cells depleted for either of these proteins (Figure 5B). Thus, these data indicate that RECQ5 and BLM act in two different pathways to suppress CO formation during HR and support our hypothesis that RECQ5 promotes SDSA. It is conceivable that at low levels of DSBs in the cell, all dHJs formed as a consequence of SDSA failure (e.g. due to RECQ5 deficiency) are dissolved by the BTR complex to yield NCO products. However, at high load of DSBs, the level of dHJs formed in SDSA-deficient cells is likely to exceed the repair capacity of the BTR complex, favoring resolution to CO products. This is consistent with our finding that RECQ5-deficient cells exhibited a marked increase in SCE frequency only on exposure to CPT (Figure 5B).

## DISCUSSION

**Figure 6.**
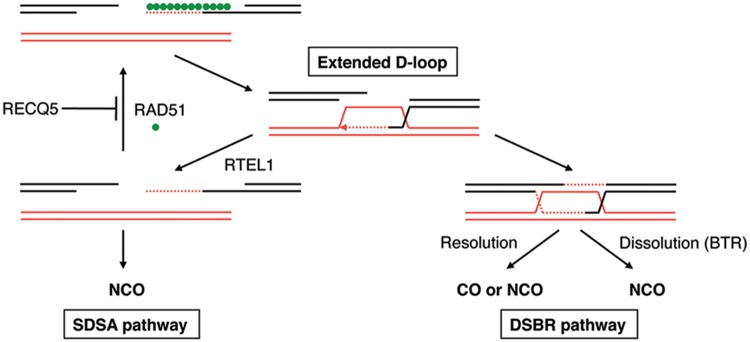
Model for the roles of RECQ5 and BLM in suppression of COs during DSBR by HR. RECQ5 promotes SDSA by disrupting aberrant RAD51-filaments formed after unwinding of the extended D-loop. BLM acts as a part of the BTR (BLM-TOPOIIIα-RMI1/2) complex to mediate dissolution of dHJs. RAD51 filaments formed during the post-synaptic phase of SDSA can promote re-invasion of the homologous duplex followed by formation of a dHJ structutre, increasing the risk of COs. RTEL1 promotes SDSA by catalyzing D-loop disruption. SDSA, synthesis-dependent strand annealing; dHJ, double Holliday junction; and DSBR, double-strand break repair.

Here we provide several lines of evidence suggesting that the human RECQ5 helicase promotes DNA DSBR by the SDSA pathway of HR. We show that (i) RECQ5 promotes the formation of NCO products during DSB-induced HR and counteracts the inhibitory effect of RAD51 filaments on DSBR by SSA; (ii) RECQ5 helicase alleviates the inhibitory effect of RAD51-ssDNA filament on RAD52-mediated DNA annealing *in vitro*; (iii) RECQ5 depletion in human cells leads to increased occupancy of RAD51 at chromatin flanking a DSB; and (iv) RECQ5 depletion is associated with increased frequency of SCEs in cells lacking the dHJ dissolvasome or in normal cells on induction of a high load of DNA damage. These data suggest that RECQ5 might act as a RAD51 filament disruptase during the post-synaptic phase of SDSA to prevent reformation of the D-loop, which would favor the classical DSBR pathway and hence increase the risk of COs (Figure 6). Futile cycles of D-loop disruption and reformation could also lead to cell death due to persistence of recombination intermediates. Consistent with our hypothesis, it has been shown that genetic ablation of RECQ5 in mouse cells is associated with persistence of RAD51 foci and increased lethality after exposure of cells to CPT, which induces DSBs during replication (20,36). Moreover, ablation of the interaction between RECQ5 and RAD51 leads to an elevation of SCEs in chicken DT40 cells lacking BLM (24). Our hypothesis is also supported by the finding that overexpression of human RAD51 has a dominant negative effect on DSB-induced gene conversion in CHO and human cells (37). RAD51 overexpression can also stimulate SCEs and the formation of interchromosomal COs (38,39). Thus, our study establishes RECQ5 as a factor that controls HR sub-pathway choice.

Counteracting Rad51 filament formation during post-synaptic phase of SDSA could as well be the underlying mechanism for the anti-recombinase function of Srs2 in budding yeast. Consistent with this proposal, it has been shown that overexpression of Rad51 in Δ*srs2* mutant cells reduces cell survival on DSB induction and nearly eliminates the NCO pathway without affecting the formation of COs, providing evidence that Rad51 inhibits the post-synaptic stage of SDSA (14,40). In addition, like RECQ5, Srs2 counteracts the inhibitory effect of Rad51 on DSBR by SSA (14,41–43). Moreover, it has been shown that overexpression of Srs2 suppresses the high level of COs in Δ*sgs1* cells, suggesting that Srs2 can shift the balance between DSBR and SDSA pathways in favor of the latter (14). This is further supported by the observation that Δ*srs2* cells show increased frequency of CO events during allelic recombination (14). Finally, yeast Rad51 was shown to inhibit Rad52-mediated DNA annealing *in vitro* (44).

It remains to be determined as to how the anti-recombinase activity of RECQ5 is regulated to prevent disassembly of the legitimate RAD51 filaments. One possibility that deserves further investigation is that the action of this helicase on RAD51 filaments might be counteracted by RAD51 mediators such as BRCA2, which facilitates filament assembly by stabilizing RAD51 binding to ssDNA (45,46). In support of this notion, it has been demonstrated that the inhibitory effect of Srs2 on Rad51 focus formation in yeast cells is antagonized by Rad52, which promotes Rad51 filament assembly by a mechanism similar to that of BRCA2 (47). Moreover, yeast Rad52 has been shown to inhibit Srs2-catalyzed Rad51-ssDNA filament disruption *in vitro* (47). It is also interesting to note that it has recently been shown that RECQ5 and BRCA2 interact with the same region on RAD51, suggesting that they exert their effect on RAD51 in a mutually competitive manner (24). In addition, overexpression of RECQ5 has been shown to impair HR-mediated repair of I-SceI-induced DSB in the HEK293/DR-GFP cells, suggesting that high levels of RECQ5 inhibit HR at the step of presynaptic RAD51 filament assembly by outcompeting the action of RAD51 mediators (21).

A previous study has shown that deletion of the mouse RECQ5 gene results in an increased frequency of I-SceI-induced HR events in cells carrying an *SCneo* reporter cassette (20). However, it has been reported that in addition to NCO-associated gene conversion, a functional *Neo* gene in this reporter system is generated by a CO recombination event, which might increase in frequency in absence of RECQ5 (48). Similarly, the frequency of I-SceI-induced HR repair within an integrated *SCneo* substrate was significantly elevate on depletion of RTEL1, which is proposed to promote SDSA by catalyzing D-loop disruption (49,50).

Disruption of the RECQ5 gene in mice leads to elevated levels of chromosomal rearrangements and cancer susceptibility (20). Moreover, a recent study has shown that RECQ5 expression levels are significantly reduced in primary colorectal cancer cells (51). Our findings suggest that these phenotypes may be a consequence of deregulation of RAD51 filament assembly, which may alter recombination pathways, leading to genomic instability. Interestingly, increased expression of the RAD51 protein has been reported in immortalized and tumor cells and its link to the genomic instability observed in these cells has been established (39,52). Thus, our study provides further insight into the molecular mechanisms underlying the build up of genomic instability associated with tumor progression.

## SUPPLEMENTARY DATA

Supplementary Data are available at NAR Online, including [21,30,35,51–59].

Supplementary Data
