# Supplementary material for: Human RECQ5 helicase promotes repair of DNA double-strand breaks by synthesis-dependent strand annealing
Source: Nucleic Acids Res. 2013 Dec 5;42(4):2380–90. doi: 10.1093/nar/gkt1263 (PMC3936725; doi:10.1093/nar/gkt1263)
Supplement: Supplementary Data [file supp_42_4_2380__index.html]

Human RECQ5 helicase promotes repair of DNA double-strand breaks by synthesis-dependent strand annealing — Human RECQ5 helicase promotes repair of DNA double-strand breaks by synthesis-dependent strand annealing — Supplementary Data 

# Human RECQ5 helicase promotes repair of DNA double-strand breaks by synthesis-dependent strand annealing

## Supplementary Data

files

**Files in this Data Supplement:**

- Supplementary Data - pdf file
