# Supplementary material for: Human RECQ5 helicase promotes repair of DNA double-strand breaks by synthesis-dependent strand annealing
Source: Nucleic Acids Res. 2013 Dec 5;42(4):2380–90. doi: 10.1093/nar/gkt1263 (PMC3936725; doi:10.1093/nar/gkt1263)
Supplement: Supplementary Data [file supp_gkt1263_nar-02448-d-2013-File008.pdf]

Supplementary Information

Supplementary Materials and Methods

Antibodies and chemicals

Rabbit polyclonal anti-RECQ5 and anti-RAD51 antibodies used for Western blotting were made in the laboratory. Control IgG used for ChIP was purified from a rabbit preimmune serum. The following commercial antibodies were used in this study: rabbit polyclonal anti-BLM antibody was purchased from Abcam; rabbit polyclonal anti-TFIIH, anti-RAD51 (used for ChIP) and anti-RAD52 antibodies, goat polyclonal Omni-probe antibody and mouse monoclonal anti-β-tubulin were purchased from Santa Cruz. Chemicals used were purchased either from Sigma-Aldrich or Roche.

DNA oligonucleotides

DNA oligonucleotides used for strand-annealing assays were purchased PAGE-purified from Microsynth. The sequences of oligonucleotides used were the following:
f9 (59-mer):
5′ACTATCATTCAGTCATGTAACCTAGTCAATCTGCGAGCTCGAATTCACTGGAGTGA CCT-3′
f7 (30-mer, complementary to the 5′-half of f9):
5′-ATTGACTAGGTTACATGACTGAATGATAGT-3′
f9-C (59-mer, fully complementary to f9):
5′-AGGTCACTCCAGTGAATTCGAGCTCGCAGATTGACTAGGTTACATGACTGAATGA TAG-3′.
Primers used in ChIP-qPCR assays were the following:
<u>DR-GFP-P1</u>
Forward: 5′-TCTTCTTCAAGGACGACGGCAACT-3′
Reverse: 5′-TTGTAGTTGTACTCCAGCTTGTGC-3′
<u>DR-GFP-P2</u>
Forward: 5′-ACGAGAGATCTATAGATCTATAGATCATGA-3′
Reverse: 5′-CTCTTTACTGAAGGCTCTTTA-3′

Protein purification

Human RECQ5, RECQ5K58R, RECQ5$^{\Delta 652\text{-}674}$, RAD51, RAD51^{K133R} and RPA were over-produced in bacteria and purified as described previously (1-4). Human RAD52 was over-produced in *E. coli* BL21 (DE3) pLysS as a fusion with a hexahistidine (6xHis) tag, and purified to homogeneity as described previously with minor modifications (5). Briefly, harvested cells were resuspended in 40 ml of buffer N [20 mM Tris-HCl (pH 8.0), 0.5 M NaCl, 10% (v/v) glycerol, 0.02% (v/v) Triton X-100] containing 5 mM imidazole and lysed by sonication. After high-speed centrifugation, the lysate was loaded on a 5-ml Ni^{2+}-charged HiTrap Chelating HP column (GE Healthcare) equilibrated with buffer N containing 10mM imidazole. After washing with 50 mM imidazole in buffer N, 6xHis-RAD52 was eluted with a 50-500 mM imidazole gradient in buffer N. Peak fractions were pooled, diluted five times with buffer H [20 mM Tris-HCl (pH 8.0), 1 mM EDTA, 0.5 mM DTT, 10% (v/v) glycerol) and loaded onto a 5-ml HiTrap Heparin HP column (GE Healthcare) equilibrated with buffer H containing 100 mM KCl. After

washing with equilibration buffer, 6xHis-RAD52 was eluted with a 0.1-1.0 M KCl gradient in buffer H. Peak fractions were diluted twice with buffer H and aliquots were stored at -70 °C. WRN-6xHis and GST-FBH1-6xHis were produced in Sf9 insect cells and purified as previously described (6,7).

Cell culture

All cells lines used in this study were cultured in Dulbecco's modified Eagle medium (DMEM, Gibco) supplemented with 10% fetal calf serum (FCS, Gibco) and 100 U/ml penicillin/streptomycin.

Cell Cycle Analysis

For cell cycle analysis, cells were collected, washed with PBS and fixed with 70% ethanol. Next, cells were washed with PBS, treated with 0.5 mg/ml RNaseA for 30 min at RT and stained with 50 μg/ml propidium iodide. The distribution of cell cycle phases with different DNA content was determined using LSRII (BD Biosciences) and FlowJo software.

siRNA transfection

Unless indicated otherwise, all the siRNAs used in the study were purchased from Microsynth. Transfection of siRNA was carried out using Lipofectamine RNAimax (Invitrogen) according to manufacturer's instructions. The sense strand sequences of all siRNAs used are indicated below:
siLuc: 5′-CGU ACG CGG AAU ACU UCG A dTdT-3′
siRAD51: 5′-AAG GGA AUU AGU GAA GCC AAA dTdT-3′
siBRCA2: 5′-CAGGACACAAUUACAACUAAAdTdT-3′
siRAD52: 5′-AAG GAU GGU UCA UAU CAU GAA dTdT-3′
siRECQ5#1: 5′-CAG GAG GCU GAU AAA GGG UUA dTdT-3′
siRECQ5#2: 5′-GGA GAG UGC GAC CAU GGC U dTdT-3′
siFBH1: 5′-GGG AUG UUC UUU UGA UAA AdTdT-3′ (8)
siBLM#1: 5′-CCG AAU CUC AAU GUA CAU AGA dTdT-3′
siBLM#2: a smartpool siRNA from Dharmacon (a kind gift from Dr. Pietro Pichierri).

Western blotting

Whole-cell extracts were prepared as described previously with minor modifications (9). Briefly, cells were extracted using buffer W [50 mM Tris-HCl (pH 7.5), 120 mM NaCl, 0.5% (v/v) NP-40, 20 mM NaF, 15 mM sodium pyrophosphate, 1 mM EDTA, 6 mM EGTA] supplemented with 0.1 mM PMSF and protease inhibitor cocktail (cOmplete, EDTA-free; Roche). After incubation on ice for 10 min, the lysate was sonicated and clarified by centrifugation. Total protein concentration was estimated by Bradford method. For Western blotting, whole cell extracts (50 μg) were separated on either 8% or 12% SDS-polyacrylamide gel. Proteins were transferred to PVDF membrane (Hybond, GE Healthcare), probed with appropriate antibodies and immune complexes were visualized using ECL system (Pierce).

Sister chromatid exchange assay

SCE assay was done as described previously with minor modifications (10). Briefly, cells were seeded in a 6-cm plate, and two rounds of transfection with appropriate siRNAs were performed at 24 h and 48 h post seeding. Cells were then grown for 40 h in the presence of 100 μM 5-bromo-2'-deoxyuridine (BrdU), and further incubated for 2 h with 0.2 μg/ml colcemid. Camptothecin (CPT; 40 nM) was added 20 h prior to cell harvest where indicated. Metaphase cells were harvested by mitotic shake-off, swollen in 75 mM KCl for 15 min, fixed with Carnoy's buffer (3:1 methanol and glacial acetic acid), spread on a clean glass slide and air-dried. The slides were then stained with Hoechst 33258 (50 μg/ml) for 30 min, rinsed with PBS, UV (254 nm, Stratalinker) exposed for 10 min and incubated in 2x SSC for 60 min. The slides were finally stained with 7% Giemsa solution for 15 min, washed twice with water and examined under light microscope. All quantifications were carried out blind and 50 metaphases were analyzed from each condition. Data were plotted using Prism 6 software as mean with SD.

Supplementary References

1. Garcia, P.L., Liu, Y., Jiricny, J., West, S.C. and Janscak, P. (2004) Human RECQ5beta, a protein with DNA helicase and strand-annealing activities in a single polypeptide. *EMBO J*, 23, 2882-2891.
2. Schwendener, S., Raynard, S., Paliwal, S., Cheng, A., Kanagaraj, R., Shevelev, I., Stark, J.M., Sung, P. and Janscak, P. (2010) Physical interaction of RECQ5 helicase with RAD51 facilitates its anti-recombinase activity. *J Biol Chem*, 285, 15739-15745.
3. Sigurdsson, S., Van Komen, S., Petukhova, G. and Sung, P. (2002) Homologous DNA pairing by human recombination factors Rad51 and Rad54. *J Biol Chem*, 277, 42790-42794.
4. Henricksen, L.A., Umbricht, C.B. and Wold, M.S. (1994) Recombinant replication protein A: expression, complex formation, and functional characterization. *J Biol Chem*, 269, 11121-11132.
5. Benson, F.E., Baumann, P. and West, S.C. (1998) Synergistic actions of Rad51 and Rad52 in recombination and DNA repair. *Nature*, 391, 401-404.
6. Orren, D.K., Brosh, R.M., Jr., Nehlin, J.O., Machwe, A., Gray, M.D. and Bohr, V.A. (1999) Enzymatic and DNA binding properties of purified WRN protein: high affinity binding to single-stranded DNA but not to DNA damage induced by 4NQO. *Nucleic Acids Res*, 27, 3557-3566.
7. Simandlova, J., Zagelbaum, J., Payne, M.J., Chu, W.K., Shevelev, I., Hanada, K., Chatterjee, S., Reid, D.A., Liu, Y., Janscak, P. *et al.* (2013) FBH1 disrupts RAD51 filaments in vitro and modulates homologous recombination in mammalian cells. *J Biol Chem*, *in press*.
8. Fugger, K., Chu, W.K., Haahr, P., Nedergaard Kousholt, A., Beck, H., Payne, M.J., Hanada, K., Hickson, I.D. and Storgaard Sorensen, C. (2013) FBH1 co-operates with MUS81 in inducing DNA double-strand breaks and cell death following replication stress. *Nat Commun*, 4, 1423.
9. Kanagaraj, R., Saydam, N., Garcia, P.L., Zheng, L. and Janscak, P. (2006) Human RECQ5beta helicase promotes strand exchange on synthetic DNA structures resembling a stalled replication fork. *Nucleic Acids Res*, 34, 5217-5231.
10. Bayani, J. and Squire, J.A. (2005) Sister chromatid exchange. *Curr Protoc Cell Biol*, Chapter 22, Unit 22 27.

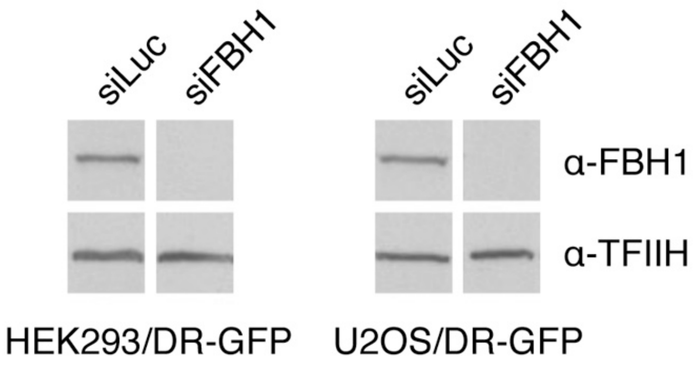

Figure S1. Confirmation of FBH1 knockdown. HEK293/DR-GFP and U2OS/DR-GFP cells were treated with indicated siRNAs. Cell extracts were subjected to Western blot analysis. Blots were probed with indicated antibodies.

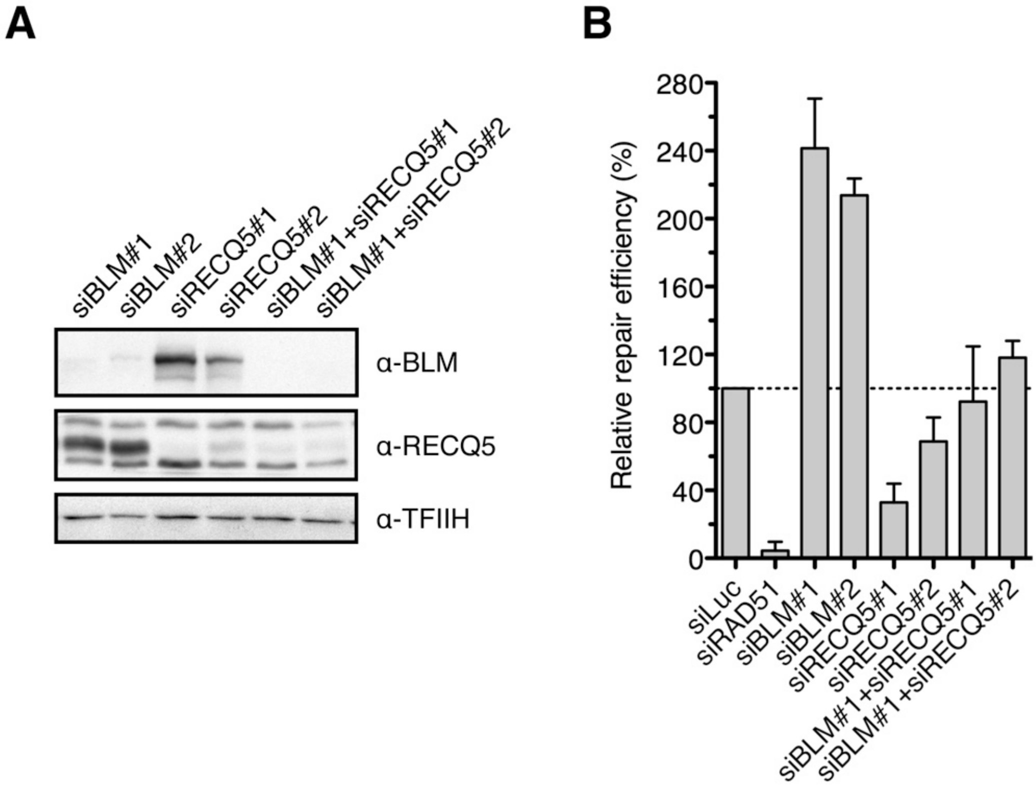

Figure S2. (A) Western blot analysis of extracts from HEK293/DR-GFP transfected with indicated siRNAs. Blots were probed with indicated antibodies. **(B)** Efficiency of HR-mediated repair of I-SceI-induced DSB in HEK293/DR-GFP cells treated with indicated siRNAs. Cells were transfected with appropriate siRNA (40 nM) two days prior to transfection of I-SceI-expressing plasmid. Percentage of GFP positive cells was measured by flow cytometry two days after DSB induction and taken as a measure of DSB repair efficiency. Values plotted represent relative repair efficiency calculated as a percentage of repair efficiency measured in cells transfected with control siRNA (siLuc; 100%). All data points represent an average of at least three replicates with error bars indicating standard deviation.

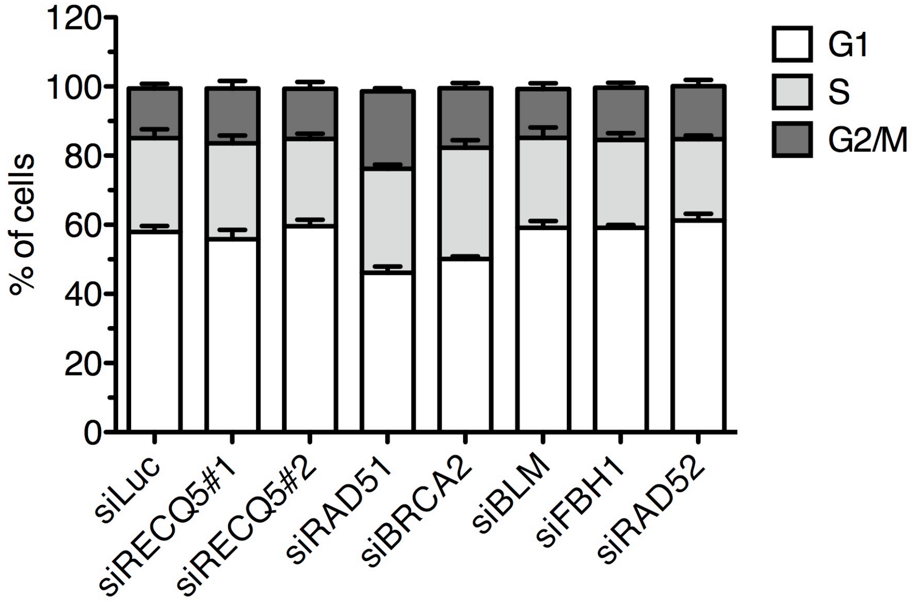

Figure S3. Cell cycle distribution of HEK293/DR-GFP cells transfected with indicated siRNAs. siRNA transfections and flow cytometry analysis were carried out as described under Materials and Methods. All data points represent an average of three replicates with error bars indicating standard deviation.

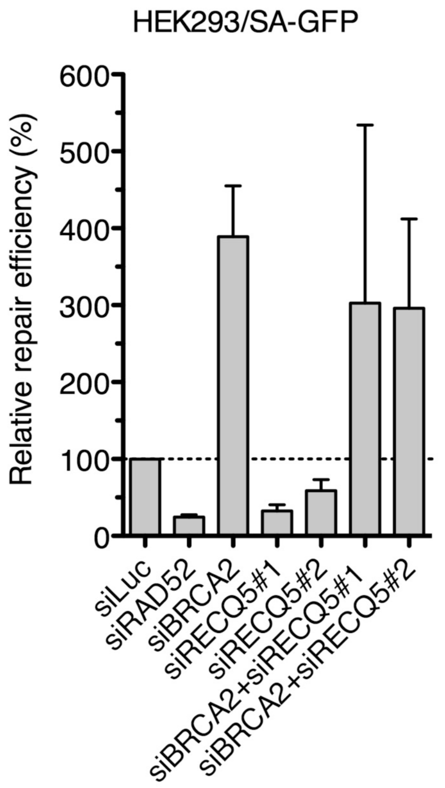

Figure S4. SSA defect of RECQ5-deficient cells is rescued by BRCA2 depletion. **(A)** Efficiency of SSA-mediated repair of I-SceI-induced DSB in HEK293/SA-GFP cells transfected with indicated siRNAs. SSA reporter assays were performed as described under Materials and Methods.

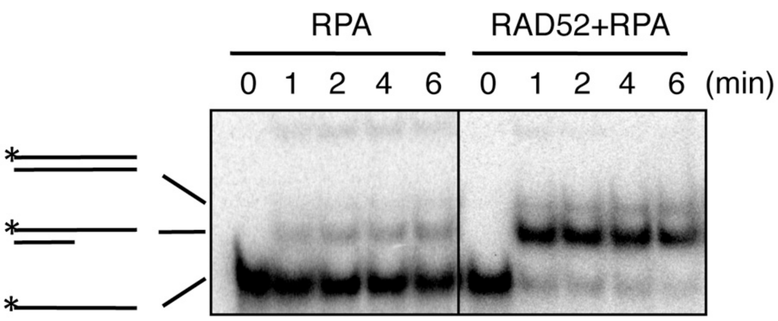

Figure S5. RAD52-mediated DNA annealing. Two complementary oligonucleotides, 5′-end radiolabeled 59mer (f9) and 30mer (f7), at a concentration of 2.5 nM, were incubated for 5 minutes in presence of RPA (30 nM) and a homologous 59mer duplex (2.5 nM). This was followed by addition of RAD52 (60 nM) where indicated. Reaction aliquots were collected at indicated time points and analyzed as in Figure 3.

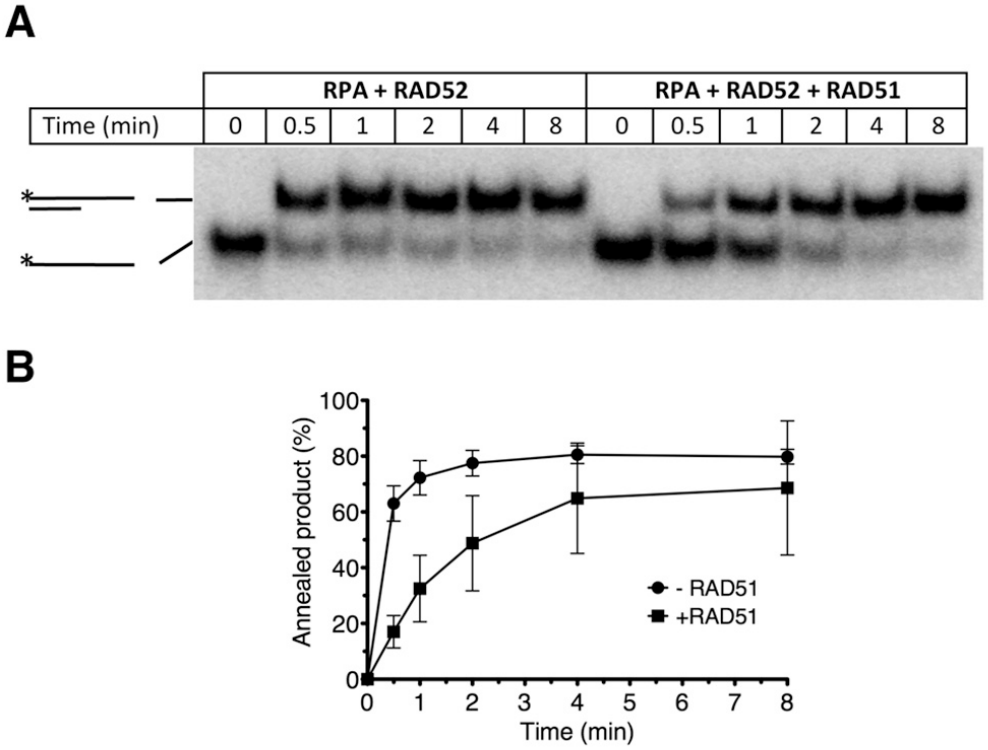

Figure S6. RAD51 inhibits RAD52-mediated ssDNA annealing *in vitro*. **(A)** Annealing reactions were carried out at 30˚C in buffer R supplemented with ATP-regenerating system. Reactions contained 5′-end radiolabeled 59-mer oligonucleotide (2.5 nM), either free or pre-coated with wild-type RAD51 (300 nM), a 30-mer oligonucleotide (2.5 nM) complementary to the 5′-half of the 59-mer, RAD52 (60 nM) and RPA (30 nM). Reaction aliquots at indicated time points were subjected to PAGE followed by phosphorimaging as described in Materials and Methods. (**B**) Quantification of data shown in (A). Each data point represents the mean of three independent experiments. Error bars represent standard deviation.

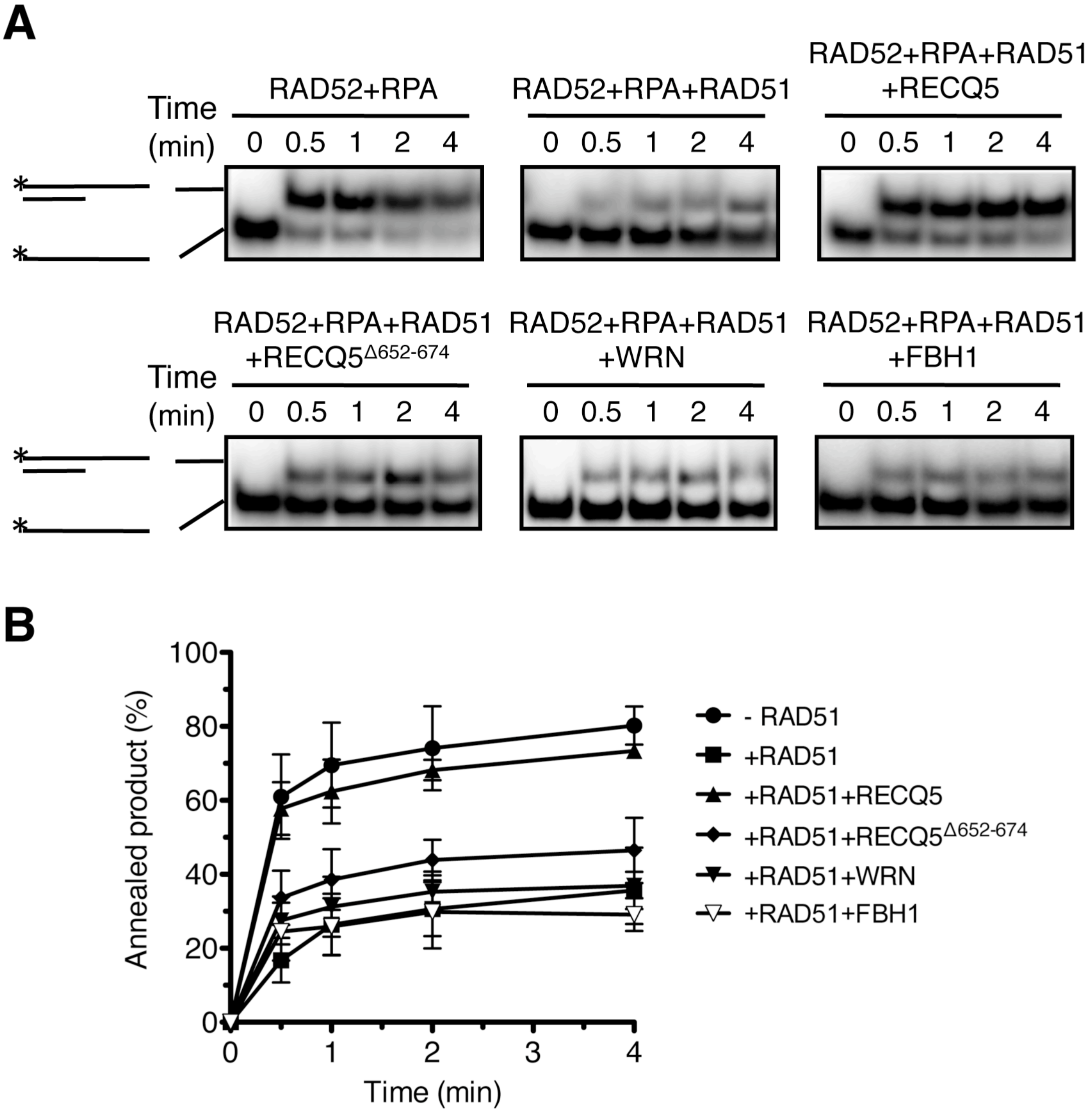

Figure S7. RECQ5, but not WRN or FBH1, alleviates the inhibitory effect of RAD51^{K133R} on RAD52-mediated ssDNA annealing. (**A**) Reactions were carried out at 30˚C in buffer R supplemented with ATP-regenerating system. Reactions contained 5′-end radiolabeled 59-mer oligonucleotide (2.5 nM), either free or pre-coated with RAD51^{K133R} (100 nM), a 30-mer oligonucleotide (2.5 nM) complementary to the 5′-half of the 59-mer, RAD52 (60 nM) and RPA (30 nM). Where indicated, RECQ5, RECQ5$^{\Delta 652\text{-}674}$, WRN and FBH1 were present at a concentration of 40 nM. Reaction aliquots at indicated time points were subjected to PAGE followed by phosphorimaging as described in Materials and Methods. (**B**) Quantification of data shown in (A). Each data point represents the mean of two independent experiments. Error bars represent standard deviation.
